# 176. Impact of Rapid Blood Culture Identification Panel on Antibiotic Treatment and Clinical Outcomes in Bloodstream Infections, Particularly Those Associated with Multidrug-resistant Microorganisms

**DOI:** 10.1093/ofid/ofad500.249

**Published:** 2023-11-27

**Authors:** Ji Yun Bae, Jiyeon Bae, Min-Kyung So, Hee Jung Choi, Miae Lee

**Affiliations:** Ewha Womans University College of Medicine, Seoul, Seoul-t'ukpyolsi, Republic of Korea; Ewha Womans University College of Medicine, Seoul, Seoul-t'ukpyolsi, Republic of Korea; Ewha Womans University College of Medicine, Seoul, Seoul-t'ukpyolsi, Republic of Korea; Ewha Womans University College of Medicine, Seoul, Seoul-t'ukpyolsi, Republic of Korea; Ewha Womans University College of Medicine, Seoul, Seoul-t'ukpyolsi, Republic of Korea

## Abstract

**Background:**

The FilmArray Blood Culture Identification (BCID) panel (BioFire Diagnostics, USA) can detect 19 bacteria, five *Candida spp.,* and three antimicrobial resistance determinants (mecA, vanA/B, and bla-KPC). This study evaluated the impact of BCID on the time to effective antibiotic administration and clinical outcomes in bloodstream infections, particularly carbapenem-resistant Enterobacterales (CRE), vancomycin-resistant enterococci (VRE) and carbapenem-resistant *Acinetobacter baumannii* (CRAB) bacteremia.

**Methods:**

The clinical outcomes and time to effective antibiotic administration for bloodstream infections were retrospectively compared between patients who had undergone BCID testing and conventional culture method (control group). Further, we compared the 30-day mortality and time to effective antibiotic administration between BCID and conventional methods in CRE, CRAB, and VRE bacteremia.

**Results:**

We compared 144 and 214 patients who had undergone BCID testing and the conventional culture method, respectively. The median time until the administration of effective antibiotics (3 h for both BCID and conventional culture, *P* = 0.789), appropriate antibiotics (BCID: 37 h vs. conventional culture: 44 h, *P* = 0.727), and 30-day mortality (BCID: 9.7% vs. conventional culture: 10.7%, *P* = 0.755) did not differ significantly between the groups. Multivariable analysis revealed that BCID was not associated with 30-day mortality after adjusting for Pitt bacteremia and Charlson comorbidity index (adjusted OR: 0.833, CI: 0.398–1.743) (Table 1).

BCID did not reduce the risk of 30-day mortality in any subgroup: patients with ICU stay, hematologic department admissions, hospital-acquired infections, gram-positive and gram-negative bacteremia. Time to effective antibiotic administration was significantly reduced on using BCID in patients with CRE and VRE bacteremia, while no difference was observed in patients with CRAB bacteremia (Table 2).

**Figure:**
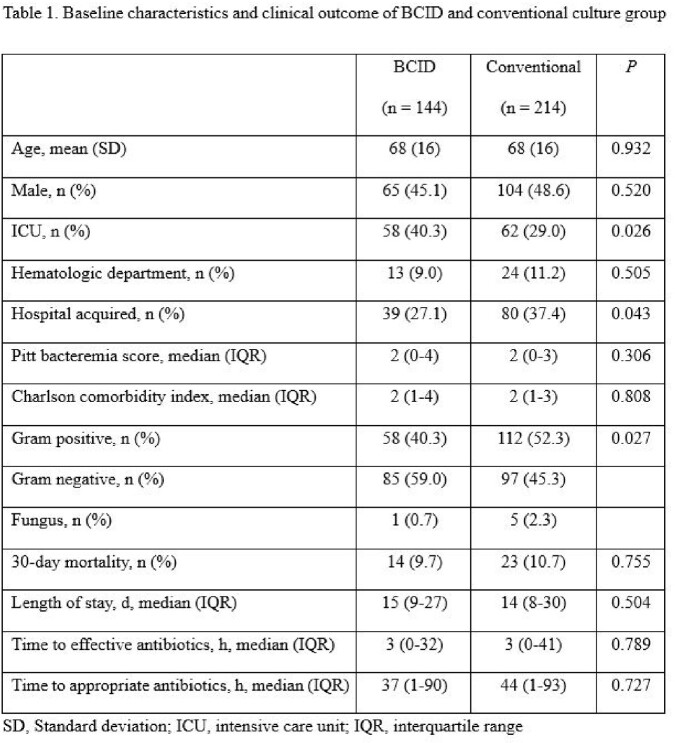

**Figure:**
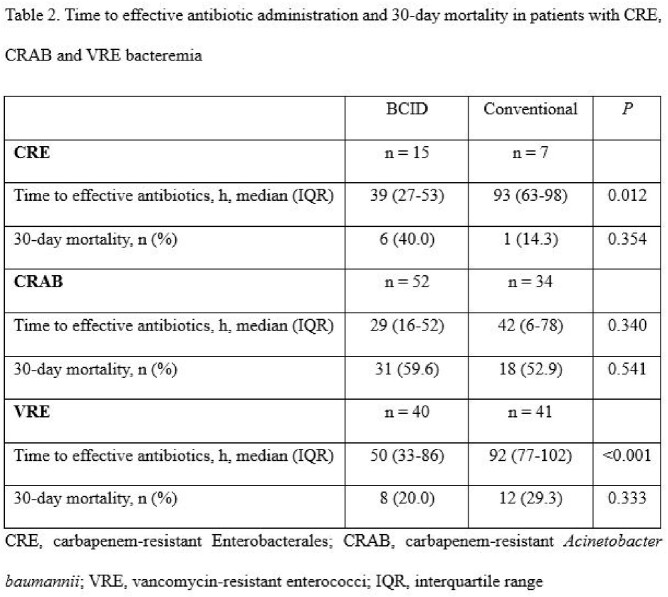

**Conclusion:**

BCID did not improve the clinical outcomes of overall bloodstream infections. However, it contributed to the early administration of effective antibiotics in patients with CRE and VRE bacteremia. BCID may be useful in selected patients in whom infection with multidrug-resistant microorganism is suspected.

**Disclosures:**

**All Authors**: No reported disclosures

